# Supplementary material for: Circulating small non-coding RNAs associated with age, sex, smoking, body mass and physical activity
Source: Sci Rep. 2018 Dec 5;8:17650. doi: 10.1038/s41598-018-35974-4 (PMC6281647; doi:10.1038/s41598-018-35974-4)
Supplement: Supplementary file 1 — Supplementary file 2 [file 41598_2018_35974_MOESM1_ESM.docx]

Supplementary File 2:

# Circulating small non-coding RNAs associated with age, sex, smoking, body mass and physical activity

###

### **Authors:**

Trine B Rounge, Sinan U Umu, Andreas Keller, Eckart Meese, Giske Ursin, Steinar Tretli, Robert Lyle, Hilde Langseth

Figure S1: Age distribution of donors shown as A) frequency plot of the continuous variable, B) boxplot of the categorical variable and C) age at donation for the blood donor groups.

Figure S2: Heatmaps of the hierarchical clustering of -log10 p-values after adjusting for age. The p-values were adjusted for multiple testing from the associations between sncRNAs from the classes miRNAs, isomiRs, tRNAs, tRNA fragments, piRNAs, lncRNAs, miscRNAs, snRNAs and mRNA fragments and the attributes blood donor group (BDg), sex, body mass, smoking (current vs never smokers) and physical activity (low vs high activity). sncRNAs are visualized if any of the associations produced p-values <0.01. Colors are yellow to orange for -log10 p-value 0 to 5 and red for -log10 p-values > 6.


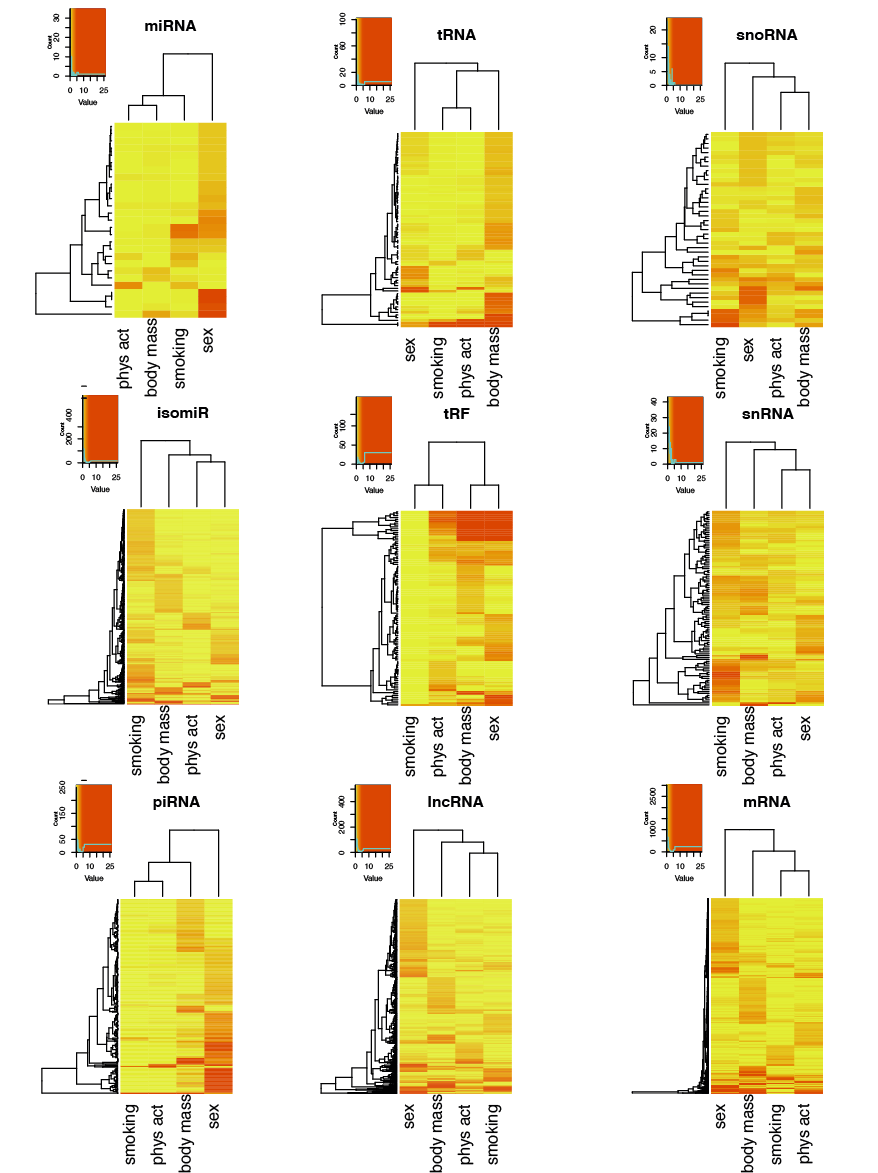


Figure S3: Volcano plots showing differential expression in log_2_fold change on the x-axis and adjusted p-values from the associations in -log10 on the y-axis. The analyses were adjusted for age. Associations with miRNAs, isomiRs, tRNAs, tRNA fragments, piRNAs, lncRNAs, miscRNAs, snRNAs and fragments mapping mRNA and the traits; blood donor group (BDg), sex, body mass smoking (current vs never smokers) and physical activity (low vs high activity) are shown.


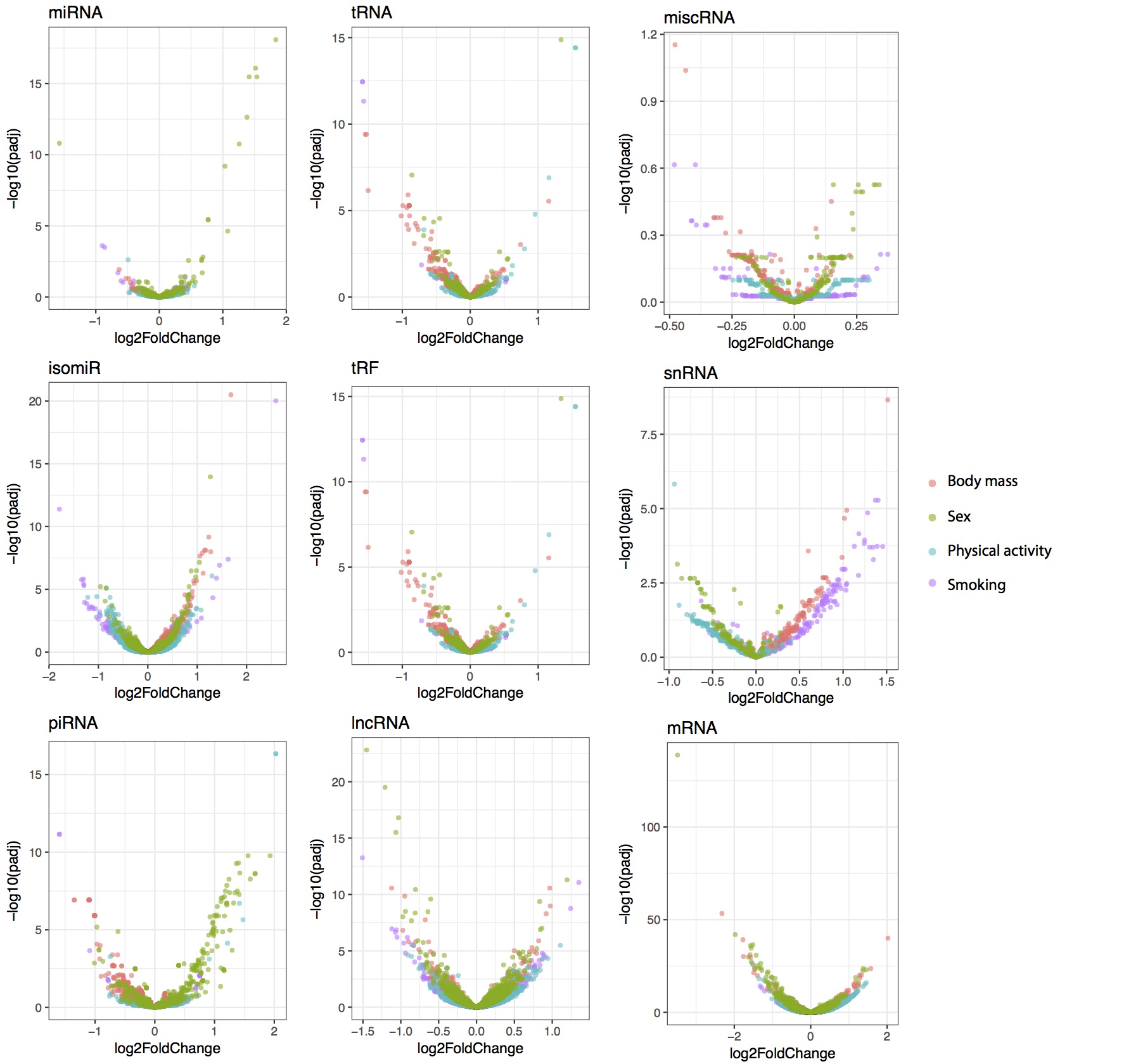


Figure S4: Volcano plots showing differential expression with age in log_2_fold change on the x-axis and adjusted p-values from the associations in -log10 on the y-axis. Associations with miRNAs, tRNAs, piRNAs and lncRNAs and age were analysed using age as categorical variable (pink), age as categorical variable using donors with blood donor group data available (green), age as categorical variable adjusted for blood donor group (light blue), age as continuous variable (dark blue) and age as continuous variable adjusted for blood donor group (violet) are shown.


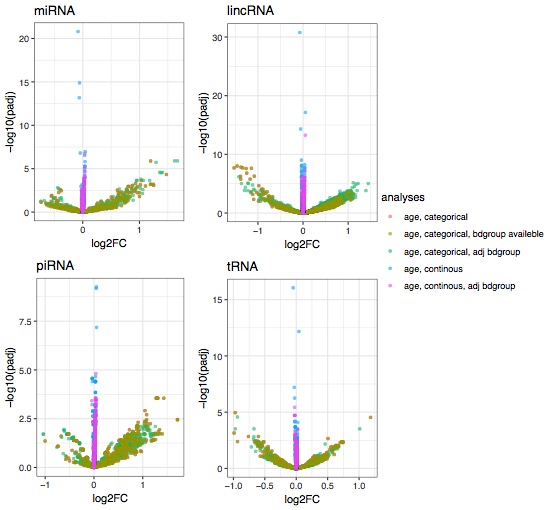


Figure S5: Weighted correlation network analysis (WGCNA) to determine co-expression modules among serum RNAs.

Figure S6: The top 3 smoking associated miRNAs relative to smoking status and dose. A) Boxplot of variance stabilized RNA counts in never former and current smokers of the top three miRNA with lowest p-values. B) Boxplots of variance stabilized RNA counts relative to the number of cigarettes smoked per day.
